# Establishment Reference Intervals Using the Kosmic Algorithm for Hemoglobin Levels and Platelet, Red Blood Cell, and White Blood Cell Counts

**DOI:** 10.1002/jcla.70341

**Published:** 2026-08-29

**Authors:** Chunyan Liu, Zhaohui Deng, Yingbo Song, Wenli Wu, Yan Li, Fang Yang, Huimin Liu, Xin Zhang

**Affiliations:** ^1^ Department of Medical Laboratory Hospital of Xinjiang Production and Construction Corps/Second Affiliated Hospital of Shihezi University School of Medicine Urumqi Xinjiang China; ^2^ Bingtuan Clinical Medical Research Center for Medical Laboratory Urumqi Xinjiang China; ^3^ Department of Clinical Laboratory Fourth Division Hospital of Xinjiang Production and Construction Corps Yining Xinjiang China; ^4^ Department of Clinical Laboratory Tumushuke General Hospital of the Third Division of Xinjiang Production and Construction Corps Tumushuke Xinjiang China

**Keywords:** hemoglobin, kosmic algorithm, platelet, red blood cell, reference interval

## Abstract

**Introduction:**

Reference intervals (RIs) for complete blood count (CBC) are crucial for assessment of overall health status and effective diagnosis of diseases. However, the RI data for CBC currently used in clinical practice in China are mainly derived from the Han Chinese population and are not stratified by ethnicity. This study aimed to calculate RIs for red blood cell count (RBC), hemoglobin (HGB), white blood cell count (WBC), and platelet count (PLT) among local populations based on ethnicity and sex in Xinjiang, China, using an indirect method to help clinicians better explain patient status in multiethnic populations.

**Methods:**

We analyzed data from 5679 individuals. We used Tukey's test to eliminate outliers based on ethnicity, sex, and age. Subgroup stratification of RIs for RBC, WBC, PLT, and HGB was determined according to a standard deviation ratio threshold. Total RIs and ethnicity‐ and sex‐specific RIs for these parameters were calculated using the kosmic algorithm.

**Results:**

The RIs for RBC and HGB must be stratified by sex, while those for PLT must be stratified by ethnicity. However, the RI for WBC does not require stratification. Sex‐stratified RIs for RBC and HGB in men showed higher upper limits than those in women. The RI for Uyghur individuals had a higher upper limit than that for Han Chinese individuals.

**Conclusions:**

Personalized RIs for CBC provide a laboratory basis for disease diagnosis and treatment and health assessment among adults in Xinjiang.

## Graphical abstract

Using kosmic algorithm and SDR analysis of real‐world data from 4795 adults in Xinjiang, we established population‐specific CBC reference intervals requiring sex stratification for RBC/HGB, ethnicity stratification for PLT, and no stratification for WBC.

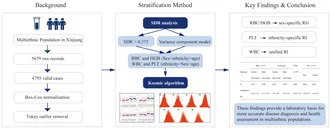

## 1 Introduction

Reference intervals (RIs) for complete blood count (CBC) are crucial for assessing overall health status and effective disease diagnosis. Shang et al. established the RIs for CBC for Han adults in China through multicenter cooperation, including parameters such as red blood cell (RBC), white blood cell (WBC), and platelet (PLT) counts and hemoglobin (HGB) level [1]. However, the RIs also depend on covariates such as ethnicity, geographic region, sex, or age and require appropriate stratification [2, 3]. Previous studies have reported differences in the RIs for CBC among ethnicities and between sexes [4], with lower RIs for HGB level in Asians than in Caucasians [5] and in women than in men [6]. Additionally, the RI for WBC count in the North China Han population is lower than that in Europeans and Mexicans in the United States [3], whereas the RI for PLT count may vary by ethnicity [7]. Therefore, the RIs for CBC stratified by ethnicity may be helpful, especially in multiethnic regions such as Xinjiang, China. However, the RI data used in clinical practice in China are mainly derived from the Han Chinese population [1], and the RIs for CBC are not stratified by ethnicity.

Currently, direct sampling is the preferred method for establishing RIs; however, this method is expensive, complicated, and difficult to implement in most laboratories [8]. Indirect methods, which combine data mining and statistical algorithms with real‐world data (RWD) to estimate RIs by identifying non‐pathological distributions, are an efficient and simple alternative for establishing RIs in the laboratory [9]. However, distinguishing non‐pathological distributions from a database of patients and healthy individuals is a key issue in establishing RIs by indirect methods. Tan et al. compared eight methods to exclude or identify pathological distribution, among which the kosmic algorithm performed the best [10].

This study aimed to identify differences in the RIs for RBC, WBC, and PLT counts and HGB level among local populations based on ethnicity and sex, as appropriately stratified RIs can help clinicians better explain patient status in multiethnic populations. Big data analysis of the physical examination findings of the adult population was performed, and the variance component model was applied to determine the necessity for dividing the RIs for CBC by ethnicity, sex, and age. The kosmic algorithm was used to establish the RIs for RBC, WBC, and PLT counts and HGB level, which provided practical experience and a theoretical reference for establishing RIs in the clinical setting.

## 2 Materials and Methods

### 2.1 Ethics Statement

This study was approved by the Ethics Committee of the Xinjiang Production and Construction Corps Hospital (approval number: 20200101) and was performed in accordance with the guidelines of the Declaration of Helsinki. As in previous studies, all participants signed informed consent [11].

### 2.2 Data Source and Preliminary Data Cleaning

This study was conducted as one of two parts of the National Clinical Key Specialty Construction Project. One part was to establish the RIs of high‐sensitivity troponin I by direct method. Another part was to utilize the data from the recruited group inspection population to establish RIs for HGB levels, as well as for PLT, RBC, and WBC counts. The first part has been published as a separate report [11].

In total, 5679 data points related to RBC, WBC, and PLT counts and HGB level were retrieved from the laboratory information system and health information management systems among the general medical population in Northern Xinjiang (Yining) and Southern Xinjiang (Tumushuke) who received physical examinations between September 2018 and March 2022. The original data were cleaned using the following screening principles to exclude data from participants (1) aged < 18 years; (2) with non‐Han or non‐Uyghur ethnicity; (3) lacking data on name, age, and RBC, HGB, WBC, and PLT measurements; and (4) who were pregnant or lactating. After cleaning, the follow‐up analysis included 4795 (84.4%) valid data points (Figure S1).

### 2.3 Quality Control

The two participating centers conducted internal quality control and external quality assurance according to the National Health Commission guidelines. RBC, HGB, WBC, and PLT values were determined using a Mindray BC‐6800 hematology analyzer (Mindray, Shenzhen, China). As previously reported [11], the measurements were performed strictly according to standard operating procedures. Quality control was performed daily at the three concentrations to ensure precision, and external quality assessments were required for each clinical laboratory to ensure accuracy. The samples were not measured until quality control was ensured. The test reagents and calibration products used in this study were provided by Mindray Laboratories.

### 2.4 Statistical Analysis

IBM SPSS Statistics for Windows, version 25.0 (IBM Corp., Armonk, NY, USA) was used to perform the statistical analyses. The RIs for RBC, WBC, and PLT counts and HGB level were established using the web‐based kosmic data mining algorithm (https://kosmic.diz.uk‐erlangen.de/) [12]. The Kolmogorov–Smirnov test was used to determine whether the data were normally distributed. Box‐Cox transformation was used to convert data to normal distributions. Tukey's test was used to identify and eliminate outlier data, where > P75 + 1.5 (P75 − P25) and < P25–1.5 (P75 − P25) were considered outliers. To ensure that the RIs for RBC, WBC, and PLT counts and HGB level did not deviate owing to differences in ethnicity, sex, and age group numbers, the Tukey method was used to identify outliers for each subgroup so that the upper and lower limits of outliers converged to a stable level without changing [13]. The dataset formed after removing outliers was statistically analyzed based on the original RBC, HGB, WBC, and PLT measures. Multiple linear regression analysis was used to determine whether HGB level and RBC, WBC, and PLT counts were influenced by ethnicity, sex, and age. In the multiple linear regression calculation, age was set as a dummy variable (18–39 years = 0,0,0; 40–59 years = 0,1,0; ≥ 60 years = 1,0,0), and Han, female individuals, and 18–39 years were defined as the reference levels for ethnicity, sex, and age, respectively. Standardized regression coefficients (*β*) were used to assess the extent to which RBC, HGB, WBC, and PLT were affected by ethnicity, sex, and age and determine the placement hierarchy of ethnicity, sex, and age in the variance component model. Furthermore, the SDR was calculated as (SD_ethnicity_, SD_sex_, or SD_age_)/SD_residual_ and was used to determine whether the RIs of RBC, HGB, WBC, and PLT must be divided by ethnicity, sex, and age. SDR > 0.375 indicated a need for subgroup division [8]. Differences with *p* < 0.05 were considered statistically significant.

## 3 Results

### 3.1 Characteristics of Data Distribution

**FIGURE 1:**
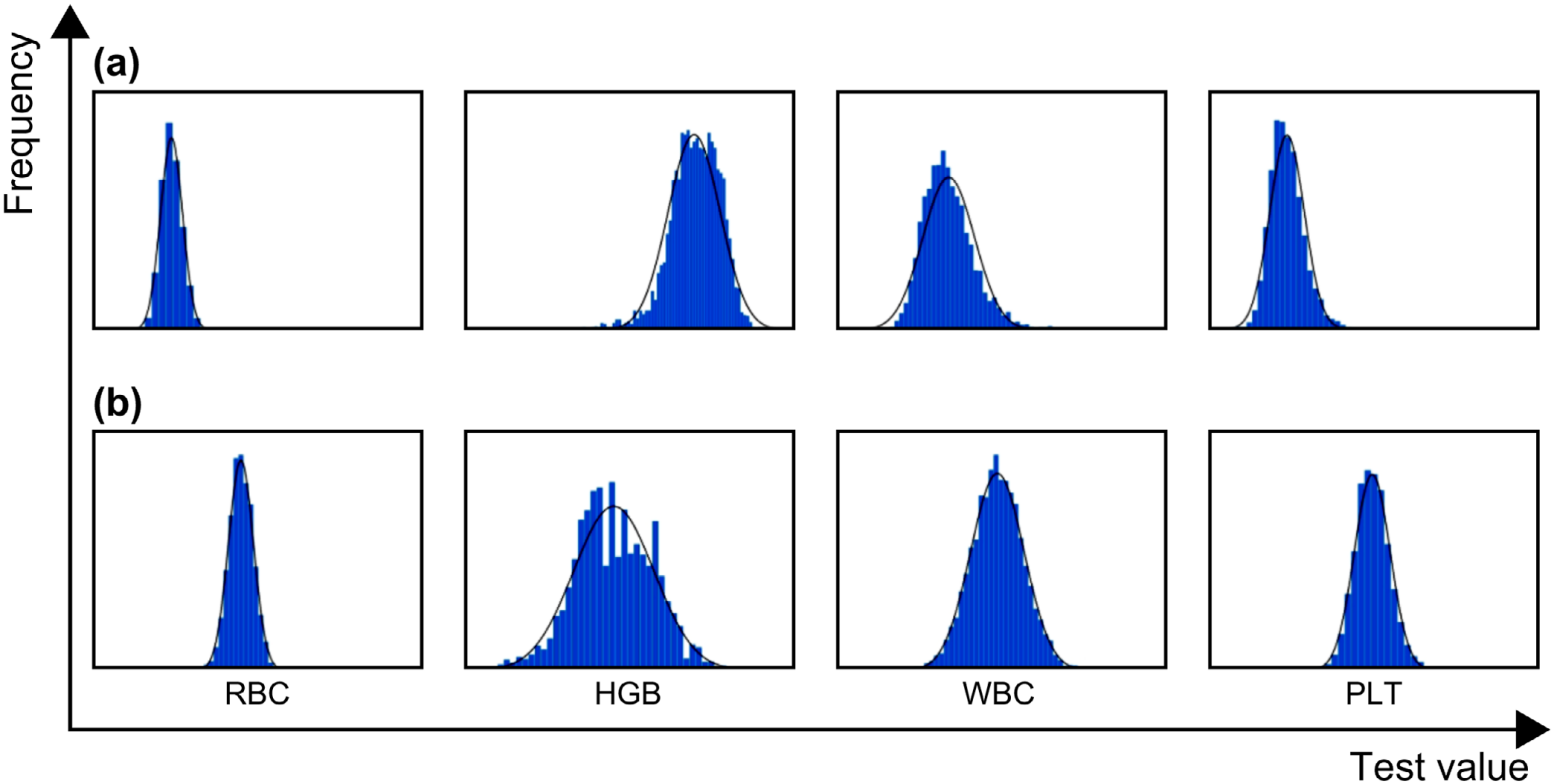
Data distributions for RBC, WBC, and PLT counts and HGB level. (a) Original distributions. (b) Distributions after Box‐Cox transformation. HGB, hemoglobin; PLT, platelet; RBC, red blood cell; WBC, white blood cell.

The RBC, HGB, WBC, and PLT data were not normally distributed. Therefore, the Box‐Cox model was used to convert the RBC, WBC, and PLT counts into normal distributions. In addition, Tukey's test was used to eliminate outliers in each group (Figure 1).

After data conversion, we found that the HGB level could not be converted to a normal value. However, the distribution of the HGB level tended to be symmetric, and the box plot was used to directly remove the outliers. The numbers of eliminated outliers are listed in Table S1.

### 3.2 Division of the RBC, HGB, WBC, and PLT Subgroups

**FIGURE 2:**
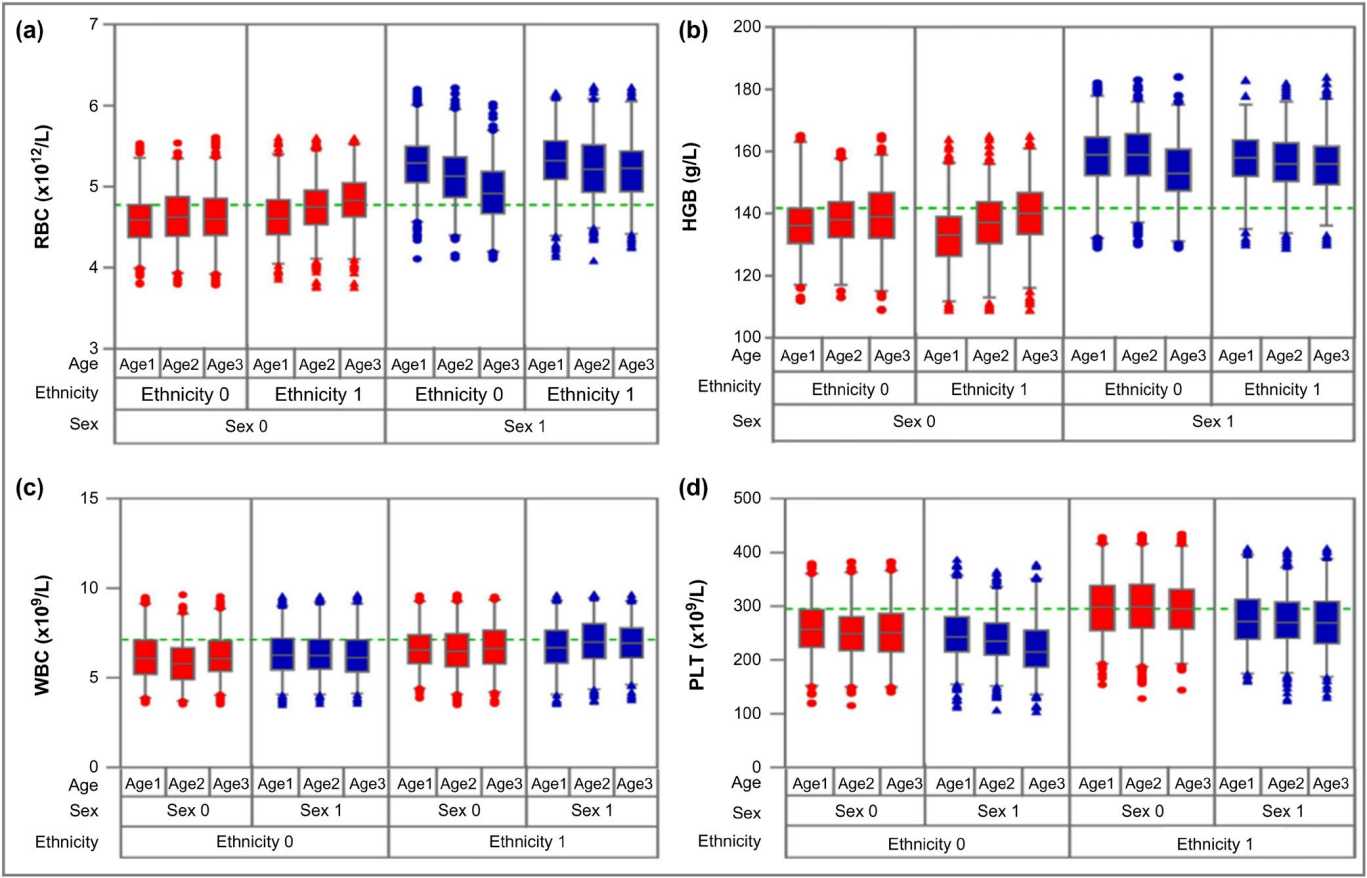
Variance component models for blood cell parameters by ethnicity, sex, and age. (a–d) Variance component models for RBC, WBC, and PLT counts and HGB level, respectively. Age 1, Age 2, and Age 3 correspond to 18–39, 40–59, and ≥ 60 years, respectively. Sex0 and Sex1 indicate female and male sex, respectively. Ethnicity0 and Ethnicity1 represent Han and Uyghur, respectively. HGB, hemoglobin; PLT, platelet; RBC, red blood cell; WBC, white blood cell.

The sex‐standardized regression coefficients for RBC count and HGB level were 0.564 and 0.675, respectively, which were greater than the value for age (Table 1). In the variance component model, sex, ethnicity, and age were placed at the first, second, and third levels, respectively (Figure 2). In contrast, the ethnicity‐standardized regression coefficients for WBC and PLT counts were 0.199 and 0.337, respectively, which were greater than those for sex and age (Table 1). Thus, ethnicity, sex, and age were placed at the first, second, and third levels, respectively (Figure 2). The standard deviation ratio (SDR) of sex for RBC count and HGB level was 0.989 and 0.959, respectively, whereas the ethnicity SDR for PLT count was 0.414 (both were > 0.375). The SDR values in the other groups were < 0.375 (Table 2). These results suggest that the RIs for RBC count and HGB level should be divided by sex, whereas that for PLT count should be divided by ethnicity.

**TABLE 1:** Standardized regression coefficients.

Group	Ethnicity		Sex		Age			
					A1		A2	
	*β*	*p*	*β*	*p*	*β*	*p*	*β*	*p*
RBC	0.136	< 0.001	0.564	< 0.001	−0.035	0.016	−0.061	< 0.001
HGB	−0.037	0.001	0.675	< 0.001	−0.032	0.015	−0.025	0.053
WBC	0.199	< 0.001	0.099	< 0.001	−0.009	0.616	0.023	0.188
PLT	0.337	< 0.001	−0.179	< 0.001	−0.036	0.023	−0.085	< 0.001

*Note:* Reference age range: 18–39 years. A1 = 40–59 years; A2 = ≥ 60 years.

Abbreviations: *β*, standardized regression coefficient; HGB, hemoglobin; PLT, platelet; RBC, red blood cell; WBC, white blood cell.

**TABLE 2:** SDRs for ethnicity, sex, and age.

Group	SD_resi_	Sex		Ethnicity		Age	
		SD	SDR	SD	SDR	SD	SDR
RBC	0.456	0.451	0.989	0.085	0.186	0.058	0.127
HGB	0.145	0.139	0.959	0.022	0.152	0.044	0.303
WBC	1.342	0.044	0.033	0.133	0.099	0.018	0.013
PLT	0.594	0.093	0.157	0.246	0.414	0.025	0.042

Abbreviations: HGB, hemoglobin; PLT, platelet; RBC, red blood cell; SD, standard deviation; SDR, standard deviation ratio; WBC, white blood cell.

### 3.3 RIs for RBC, WBC, and PLT Counts and HGB Level Determined Using the Kosmic Algorithm

**FIGURE 3:**
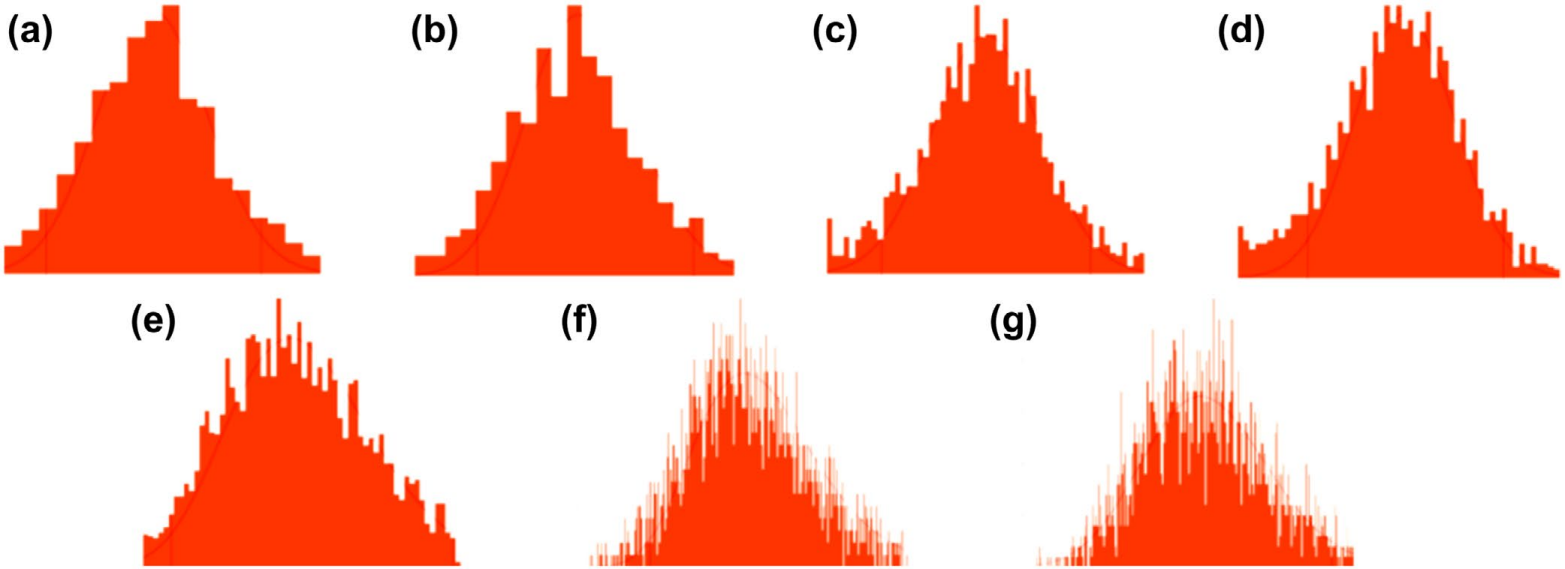
RIs calculated using the kosmic algorithm. The truncation of the parameter distribution and the observed distribution refers to the interval between the dashed lines. (a–g) Estimated distributions of the test results for RBC count (female individuals), RBC count (male individuals), HGB level (female individuals), HGB level (male individuals), WBC count, PLT count (Han), and PLT count (Uyghur). HGB, hemoglobin; PLT, platelet; RBC, red blood cell; WBC, white blood cell.

Figure 3 provides a graphical representation of the RIs determined using the kosmic algorithm, whereas Table 3 summarizes the RIs for RBC, WBC, and PLT counts and HGB levels determined using the kosmic algorithm. The upper reference limits (URLs) of the RIs for RBC count and HGB level were higher in men than in women, whereas the URL for PLT count was higher in Uyghur individuals than in Han Chinese individuals.

**TABLE 3:** RIs for RBC, WBC, and PLT counts and HGB level determined using the kosmic algorithm.

Indicator	RBC (×10^12^)		HGB (g/L)		WBC (×10^9^)	PLT (×10^9^)	
	Female individuals	Male individuals	Female individuals	Male individuals		Han	Uyghur
*N*	2365	2289	2285	2265	4463	2066	2562
2.5% percentile	4.04	4.51	119	139	4.05	158	174
50% percentile	4.64	5.18	138	158	6.38	242	285
97.5% percentile	5.26	5.93	156	176	9.55	367	413
Established RI	4.04–5.26	4.51–5.93	119–156	139–176	4.05–9.55	158–367	174–413
Existing RI	3.80–5.10	4.30–5.80	115–150	130–175	3.50–9.50	125–350	
% of individuals outside the existing RI	13.23	8.78	12.82	3.13	0.69	3.82	14.83

Abbreviations: HGB, hemoglobin; PLT, platelet; RBC, red blood cell; RI, reference interval; WBC, white blood cell.

As shown in Table 3, the RIs were compared with the existing RIs, which were derived from national reference values and are currently used in our laboratory. The upper and lower limits of WBC, RBC (both sexes), and HGB (both sexes) were all higher than those of the existing RIs. Additionally, compared with the existing RIs, both the lower and upper limits for PLT were higher in both the Han and Uyghur populations. Among all groups, the Uyghur population had the highest proportion of individuals with PLT falling outside the existing reference range (14.83%), followed by RBC (13.23% in females, 8.78% in males) and HGB (12.82% in females, 3.13% in males).

## 4 Discussion

RIs are critical in clinical diagnosis, treatment monitoring, and prognostic evaluation and are influenced by ethnicity, sex, age, region, and other factors [1, 14, 15]. Evaluating these values for CBC is critical in correctly interpreting all clinical laboratory test results. In this study, we analyzed RWD to assess the effects of ethnicity, sex, and age on RBC, WBC, and PLT counts and HGB level, using variance component models. We established RBC, HGB sex‐specific, PLT ethnicity‐specific, and WBC RIs using the kosmic algorithm. Our findings indicate that the RIs for RBC count and HGB level should be stratified by sex and the RIs for PLT count by ethnicity. Specifically, the URLs of RIs for RBC count and HGB level were higher in male individuals than in female individuals, and that of RI for PLT count was higher in the Uyghur group than in the Han Chinese group. The results of this study not only provided personalized RIs for clinicians for disease diagnosis, treatment, and health assessment but also explored the practical experience of using RWD to establish laboratory test project RIs.

To eliminate the interference of the “diseased population,” we selected participants who had undergone physical examination, whose distribution characteristics tended to match those of the community population, and whose illness had a lower proportion than that of outpatient and inpatient populations [16]. Additionally, to ensure that this study excluded diseased individuals as much as possible and given that individuals with repeated measurements were more likely to have abnormalities or diseases, we included only the data from the earliest visit records [6]. Duplicate measurements were processed in the data cleaning step, although this step had no material effect on the overall data distribution. Box‐Cox transformation was used in this study for the normality transformations of RBC, HGB, WBC, and PLT data, although the choice of data transformation method had less of an effect on the establishment of RIs for CBC than that for outlier handling and indirect algorithm selection suggested by Dan et al. [17]. The selection of outlier removal methods significantly changes the establishment of RIs for CBC, particularly when using indirect methods [18]. Owing to the difficulty in applying strict screening criteria, the calculation of RIs using the indirect method is susceptible to contamination by the “diseased population.” The Tukey method is recommended for outlier removal via the indirect method for establishing RIs, as it tends to produce narrower RIs [17, 18]. Therefore, Tukey's test was used to eliminate the outliers of RBC, WBC, and PLT data. However, as HGB data could not be converted to a normal distribution, a box plot was used to remove outliers [8].

Previous research has proposed methods on how best to divide subgroups when establishing the RIs [19]. Among these, the EP28‐A3c document discusses several possible dividing criteria, such as sex, ethnicity, age, and gestation [20]. Previous studies often evaluated the division of subgroups using multi‐factor or uni‐factor methods, in which subgroups were required for *p* < 0.05 or 0.01. However, this difference test is influenced by the sample size, and the results of the application of this method to big data analysis are often unreliable [21, 22]. Therefore, Ma et al. recommended using the SDR to determine whether RI partitions are required [8]. In the present study, the RBC and HGB SDRs of the sexes were > 0.375, indicating a sex difference [23], consistent with the findings reported by Takami et al. [6]. They reported the RI for RBC count in healthy Japanese adults and noted that the SDR for RBC count and HGB level was > 0.3, confirming that the RI should be stratified according to sex [6]. However, the ethnicity SDR for RBC count and HGB level did not exceed 0.375; therefore, subsequent calculations were not stratified by ethnicity. The ethnic SDR for PLT count was > 0.375 (0.414), suggesting that the PLT count RI should be stratified by ethnicity, which further confirms that in multiethnic areas, the PLT count RI should be divided based on ethnic subgroups [4, 13]. The present study also explored the influence of confounding factors (ethnicity, sex, and age) on CBC parameters. Therefore, the RIs for RBC count and HGB level were subsequently stratified by sex, the RI for PLT count was stratified by ethnicity, and the RI for WBC was not stratified.

Indirect methods are increasingly applied to establish RIs. Tulay et al. identified RIs for CBC in neonatal, adult, and older populations through indirect methods and confirmed that establishing RIs using data obtained from clinical laboratory databases was comparable to establishing RIs using direct methods [24]. However, no uniform criteria are available to determine the calculation method that should be applied to establish RIs for CBC using indirect methods. Smit et al. [25] used non‐parametric methods to establish these RIs for three major ethnic groups in the Western Cape region of South Africa, whereas Tulay et al. reported the use of refineR to calculate RIs for HGB level (women) and PLT count [26]. Many reports have detailed calculation methods for establishing RIs using indirect methods [9, 15, 27]. Yang et al. analyzed the accuracy of five indirect methods in establishing RIs for CBC using RWD and concluded that for data with small intra‐individual variation, the kosmic method was the most stable and least affected by data transformation and outlier processing methods [17], consistent with findings reported by Tan et al. [10]. Kosmic provides reliable RI estimates when the proportion of abnormal data is < 20%. Given that CBC parameters have narrow intra‐individual variation [4], we defined the physical examination population as the ‘reference population’ and used Tukey's test to remove outliers. This likely kept the proportion of pathological data well below the 20% threshold, thereby significantly enhancing its applicability [17]. In this study, we used the online kosmic algorithm as described by Zierk et al. [12] to establish RIs for RBC, HGB, WBC, and PLT data.

The results showed that the URLs for RIs for RBC count and HGB level in men were higher than those in women, which was consistent with the trend of RIs used in clinical laboratories in China [1]. This may also be related to chronic menstrual blood loss in women and higher androgen levels in men, resulting in higher RBC and HGB values in men than in women [1, 28]. The URL of PLT level RI in Uyghurs was higher than that in Han Chinese, Japanese adults, Korean adults, and other ethnic groups [4, 17, 29], and that of Uyghur women was significantly higher than that of Uyghur men. The use of unified PLT count RI will cause misdiagnosis of Uyghurs' PLT‐related diseases. Therefore, an ethnicity‐specific PLT count RI must be established. Although age is known to influence certain CBC parameters in some populations [29], our SDR analysis showed that age did not reach the threshold for stratification in the present study. This may be due to the relatively narrow age range of our physical examination cohort or the specific demographic characteristics of the Xinjiang population. Nonetheless, future studies with a wider age distribution and larger sample sizes are warranted to further explore potential age‐related effects.

This study has some limitations. Previous reports have shown that the sample size of RWD may be influenced by the proportion of pathological data and the degree of overlap between populations, thereby altering the RIs established using indirect methods [27]. In cases with significant disease bias in RWD, a sample size of > 5000 individuals is recommended; however, with pathological data < 30%, the sample size for kosmic to establish RIs can be as low as 1000 individuals [9]. In this study, after RBC, HGB, and PLT data were grouped by sex and ethnicity, the sample size of each subgroup was > 2000 individuals, except for the WBC data (*n* = 4463); thus, the proportion of pathological data could not be estimated. Therefore, we utilized the population with physical examination data, cleaned the data, removed outliers, and ensured that the reference population was as healthy as possible. Future research should further evaluate the clinical applicability of the new RIs by expanding the sample bank. Additionally, although we compared the newly established RIs with existing reference intervals, we did not perform clinical validation using independent disease cohorts to assess sensitivity or specificity. Such validation should be included in future studies to evaluate the clinical utility of these RIs before their widespread adoption. It is important to note that the reference intervals established in this study are specific to the analytical system used. Prior to clinical application on different platforms, method comparison and validation are necessary to ensure accuracy and reliability.

This study established RIs for RBC, WBC, and PLT counts and HGB level using indirect methods for the adult population in Xinjiang. Our findings confirmed that the RIs for RBC count and HGB level must be divided by sex, whereas that for PLT count must be divided by ethnicity. Moreover, the URLs of the RIs for RBC count and HGB level were higher in men than in women, whereas that for PLT count was higher in Uyghur individuals than in Han Chinese individuals. Establishing sex‐specific RIs for RBC count and HGB level and ethnicity‐specific RIs for PLT count may improve health assessment and the clinical application of laboratory indicators in the adult population of Xinjiang.

## Author Contributions

**Chunyan Liu:** writing – original draft. **Zhaohui Deng:** project administration. **Yingbo Song:** data curation. **Wenli Wu:** investigation. **Yan Li:** investigation. **Fang Yang:** data curation. **Huimin Liu:** data curation. **Xin Zhang:** writing – review and editing.

## Funding

This work was supported by grants from the National Clinical Key Specialty Construction Project (Bingcaishe [2023] 16 to author Chunyan Liu, Zhaohui Deng, Yingbo Song, Wenli Wu, Yan Li, Fang Yang, Huimin Liu, Xin Zhang) and the Science and Technology Research Program in Key Areas of Xinjiang Production and Construction Corps (2020AB021 [2020] to author Zhaohui Deng). The sponsors had no role in the study design; data collection, analysis, or interpretation; writing of the report; or the decision to submit the article for publication.

## Ethics Statement

This study was approved by the Ethics Committee of the Xinjiang Production and Construction Corps Hospital (approval number: 20200101) and was performed in accordance with the guidelines of the Declaration of Helsinki. All participants signed informed consent.

## Conflicts of Interest

The authors declare no conflicts of interest.

## Supporting information


**Figure S1:** Flow diagram showing the population selection criteria for estimating the RIs for RBC, WBC, and PLT counts and HGB level using indirect methods. HGB, hemoglobin; PLT, platelet; RBC, red blood cell; RI, reference interval; WBC, white blood cell.
**Table S1:** Outliers in different tests and distribution of enrolled patients according to ethnicity.

## Data Availability

The datasets generated during and/or analyzed during the current study are not publicly available due to information that could compromise the privacy of research participants.
