# Supplementary material for: Establishment Reference Intervals Using the Kosmic Algorithm for Hemoglobin Levels and Platelet, Red Blood Cell, and White Blood Cell Counts
Source: J Clin Lab Anal. 2026 Aug 29;40(19):e70341. doi: 10.1002/jcla.70341 (PMC13525422; doi:10.1002/jcla.70341)
Supplement: Supplementary file 1 [file JCLA-40-e70341-s001.docx]

**Supplementary Information**

**Supplementary Figure S1. Flow diagram showing the population selection criteria for estimating the RIs for RBC, WBC, and PLT counts and HGB level using indirect methods**. RI, reference interval; RBC, red blood cell; HGB, hemoglobin; WBC, white blood cell; PLT, platelet.

**Supplementary Table S1. Outliers in different tests and distribution of enrolled patients according to ethnicity.**

| Group | Original | Outliers | Data analyzed | | |
| --- | --- | --- | --- | --- | --- |
|  |  |  | Total | Han | Uyghur |
| RBC (×10^12^/L) | 4,795 | 141 | 4,654 | 2,052 | 2,602 |
| HGB (g/L) | 4,795 | 245 | 4,550 | 2,033 | 2,517 |
| WBC (×10^9^/L) | 4,795 | 332 | 4,463 | 2,008 | 2,455 |
| PLT (×10^9^/L) | 4,795 | 167 | 4,628 | 2,066 | 2,562 |

RBC, red blood cell; HGB, hemoglobin; WBC, white blood cell; PLT, platelet.
